# Bumped Kinase Inhibitor BKI-1708 Interferes in Cytokinesis and Drives Baryzoite Conversion in the Cyst-Forming Apicomplexan Parasites *Toxoplasma gondii*, *Neospora caninum* and *Besnoitia besnoiti*

**DOI:** 10.3390/ijms27062914

**Published:** 2026-03-23

**Authors:** Maria Cristina Ferreira de Sousa, Joachim Müller, Kai Pascal Alexander Hänggeli, Manfred Heller, Anne-Christine Uldry, Sophie Braga-Lagache, Alexandre Leitao, Luis-Miguel Ortega-Mora, Kayode K. Ojo, Wesley C. Van Voorhis, Andrew Hemphill

**Affiliations:** 1 Institute of Parasitology, Department of Infectious Diseases and Pathobiology, Vetsuisse Faculty, University of Bern, 3012 Bern, Switzerland; joachimm.mueller@unibe.ch (J.M.); kai.haenggeli@unibe.ch (K.P.A.H.); 2 Graduate School for Cellular and Biomedical Sciences (GCB), University of Bern, 3012 Bern, Switzerland; 3 Department for BioMedical Research (DBMR), Proteomics and Mass Spectrometry Core Facility, University of Bern, 3012 Bern, Switzerland; manfred.heller@unibe.ch (M.H.); anne-christine.uldry@unibe.ch (A.-C.U.); sophie.lagache@unibe.ch (S.B.-L.); 4 CIISA—Centre for Interdisciplinary Research in Animal Health, Faculty of Veterinary Medicine, University of Lisbon, 1300-477 Lisbon, Portugal; alexandre@fmv.ulisboa.pt; 5 Saluvet, Animal Health Department, Faculty of Veterinary Sciences, Complutense University of Madrid, Ciudad Universitaria S/N, 28040 Madrid, Spain; luis.ortega@ucm.es; 6 Department of Medicine, Center for Emerging and Re-Emerging Infectious Diseases, University of Washington, Seattle, WA 98109-4766, USA; ojo67kk@washington.edu (K.K.O.); wesley@uw.edu (W.C.V.V.)

**Keywords:** coccidia, apicomplexan parasites, calcium dependent protein kinase, mitogen activated protein kinase-like 1, bumped kinase inhibitor, multinucleated complexes, baryzoites, proteomics

## Abstract

Bumped kinase inhibitors (BKIs) have demonstrated safety and promising efficacy against various apicomplexan pathogens both in vitro and in vivo, but do not act parasiticidal in vitro. In the closely related cyst-forming coccidians *Toxoplasma gondii*, *Neospora caninum* and *Besnoitia besnoiti*, treatments with BKI-1708 induce the conversion of intracellular tachyzoites into atypical multinucleated complexes named “baryzoites”. In this study, we comparatively assessed tachyzoites and baryzoites of all three species with respect to ultrastructure, differential antigen expression by immunofluorescence, and overall differential protein expression by MS-proteomics. TEM demonstrated common, but also distinguishing, structural features in baryzoites of the three species. They contained newly formed zoites, unable to complete cytokinesis, and thus they were trapped intracellularly. An electron-dense cyst wall-like structure was found only in *T. gondii* baryzoites. Species-specific differences in antigen expression were observed by immunofluorescence. Comparative proteomic analysis of baryzoites versus tachyzoites revealed a downregulation of ribosomal proteins, proteins associated with secretory organelles, as well as of transcription and translation factors in baryzoites across all species. Bradyzoite-specific markers were upregulated only in *T. gondii* baryzoites. Two alveolin-domain filament proteins and a hypothetical protein (TGME49_236950, NCLIV_050850, BESB_060040) were detected at higher abundance in all three species. Thus, baryzoites exhibit distinct phenotypic and proteomic profiles, with ambiguous expression of tachyzoite and bradyzoite antigens, suggesting a reversible response to stress rather than progression into a fully differentiated form.

## 1. Introduction

The phylum Apicomplexa (within the superphylum Alveolata) includes several intracellular parasites of significant medical and veterinary importance, among which the cyst-forming coccidia *Toxoplasma gondii*, *Neospora caninum*, and *Besnoitia besnoiti* form a closely related cluster within the family Sarcocystidae [1]. The genomes of *T. gondii, N. caninum* and *B. besnoiti* exhibit a high degree of synteny and encode a large number of known and inferred orthologues that are important for critical processes such as host cell invasion, immune modulation, intracellular survival as well as sexual development [2,3,4].

*T. gondii* and *N. caninum* undergo a life cycle that alternates between intermediate and definitive hosts. The intermediate hosts harbor two stages: (i) the rapidly proliferating and disease-causing tachyzoites, which undergo continuous lytic cycles and thus inflict tissue damages and immunopathology, and (ii) the slowly proliferating bradyzoites that form long-lived tissue cysts mostly in the central nervous system and muscle tissues, representing the chronic stage of infection, which normally does not cause clinical symptoms. Within the intestine of the definitive hosts—felids in the case of *T. gondii* and canids for *N. caninum*—sexual development results in the production of oocysts that are shed with the feces, and sporulation in the environment leads to the formation of orally infective oocysts containing sporozoites [4,5]. For *B. besnoiti*, only tachyzoites and bradyzoites have been described so far. While the genes coding for proteins associated with sexual stages have been identified, sexual reproduction has not been demonstrated and the definitive host, if it exists, is not yet known [3].

*T. gondii* is distributed worldwide and capable of infecting virtually all warm-blooded animals including humans. Transmission occurs through the accidental ingestion of oocysts, or through bradyzoite containing tissue cysts in undercooked meat [6]. Vertical transmission by *T. gondii* tachyzoites occurs with maternal primary infection during pregnancy, often leading to abortion, stillbirth, or severe developmental abnormalities in the fetus [6,7], not only in humans, but also in other species such as small ruminants in particular. *T. gondii* infection can also lead to severe neurological and ocular complications, particularly in immunocompromised individuals, and congenitally infected infants. During chronic infection, *T. gondii* bradyzoites encapsulated in tissue cysts are mostly asymptomatic, but the loss of immunocompetence can lead to recrudescence, conversion into tachyzoites, and acute disease. *N. caninum* shares many features with *T. gondii* and primarily affects cattle and dogs, but infections in humans have not been reported [8]. Neosporosis in cattle can lead to abortion, birth of weak offspring, or birth of healthy calves that then transmit the parasite to the next generation. In chronically infected animals, pregnancy can lead to recrudescence, and repeated abortions can occur. As a leading cause of reproductive problems in cattle, neosporosis results in substantial economic losses to the livestock industry [4,9]. *B. besnoiti* is less well-studied, and cattle represent the main intermediate host, which suffers from acute disease caused by the rapid proliferation of tachyzoites in different tissues. The chronic stage is characterized by the presence of large tissue cysts with a multilayered cyst wall, which are formed mostly in the dermis, sclera and mucosa of affected animals. Bovine besnoitiosis is commonly associated with abortion and decreased milk production, weight loss, bull infertility and reduction in hide quality [10]. However, contradictory observations have been reported, and it is clear that there has been insufficient in-depth study on the impact of the disease on production [11]. The only mode of transmission known to date is mechanically, either through direct contact from infected animals (e.g., during natural mating), or via flies or biting insects [10]. There is no evidence for vertical transmission of *B. besnoiti* to date.

For all three species, the formation of tissue cysts in intermediate hosts is crucial for persistence and disease transmission. *T. gondii* and *N. caninum* typically form cysts in the neural or muscular tissues [5], while *B. besnoiti* forms cysts in the dermis, sclera and mucosal tissues [3]. The differentiation into persistent tissue cysts that are largely refractory to drugs is also one of the reasons why current treatment options fail to cure the diseases caused by these parasites. The first-line treatments for acute toxoplasmosis consist of pyrimethamine-sulfadiazine or trimethoprim-sulfamethoxazole that interfere in the folic acid pathway. Other treatment options include the macrolide antibiotic spiramycin (early in pregnancy) and the lincosamide clindamycin [12], or the napthoquinone atovaquone [13]. All these drugs are repurposed and lack optimal efficacy and safety profiles. No drugs have been licensed for the use against neosporosis or besnoitiosis so far, although a wide range of compounds have been repurposed in experimental settings [4,10].

Bumped kinase inhibitors (BKIs), which were either designed on a pyrazolopyrimidine (PP) or 5-aminopyrazole-4-carboxamide (AC) scaffold, have emerged as promising pre-clinical drugs for the treatment of diseases caused by a wide range of apicomplexan parasites [4,14,15]. BKIs were originally designed to selectively target calcium-dependent protein kinase 1 (CDPK1), which is conserved among apicomplexans but absent in mammalian hosts. CDPK1 is required for microneme secretion, gliding motility, host cell invasion and egress [16]. More recently it has been shown that BKIs also target *T. gondii* mitogen-activated protein kinase-like 1 (TgMAPKL-1). TgMAPKL1 plays a key role in the *T. gondii* cell cycle by regulating centrosome duplication, which is essential for proper division into two daughter cells during endodyogeny. Mutations in TgMAPKL1 can disrupt this process, leading to defects in cell division and parasite growth [17,18]. Treatments of *T. gondii*, *N. caninum* and *B. besnoiti* cultured in fibroblasts or MARC cells with a range BKIs resulted not only in impaired host cell invasion, but also in the transformation of intracellular tachyzoites into schizont-like multinucleated complexes (MNCs) [4,14,19]. These MNCs were characterized by ongoing nuclear division with incomplete cytokinesis and the presence of newly formed intracellular zoites, neither bradyzoites nor tachyzoites, which are not individualized and lack an outer plasma membrane. MNCs remained viable in vitro for extended periods of time yet remained confined to the host cell interior. Upon removal of the drug, the effect was reversible, leading to the re-emergence of tachyzoites. MNCs were named “baryzoites” from the Greek βαρύς = massive, bulky, heavy, or inert [19]. Thus, baryzoites constitute a drug-induced stage that promotes parasite survival upon prolonged exposure to elevated drug concentrations.

One of the compounds that induced the conversion of tachyzoites into baryzoites is the AC compound BKI-1708 [20]. BKI-1708 efficiently inhibited the proliferation of *T. gondii* and *N. caninum* in vitro. In experimentally infected pregnant mice, BKI-1708 treatment was safe and resulted in significantly decreased cerebral parasite loads in the dams, dramatically increased pup survival and strongly reduced vertical transmission [20]. In this study, we comparatively analyzed the BKI-1708 induced baryzoites of the closely related cyst-forming coccidians *T. gondii*, *N. caninum*, and *B. besnoiti,* highlighting similarities and species-specific differences on the ultrastructural and proteomic level.

## 2. Results

### 2.1. Ultrastructural Features of T. gondii, N. caninum and B. besnoiti Baryzoites Induced by Treatment with BKI-1708

**Figure 1:**
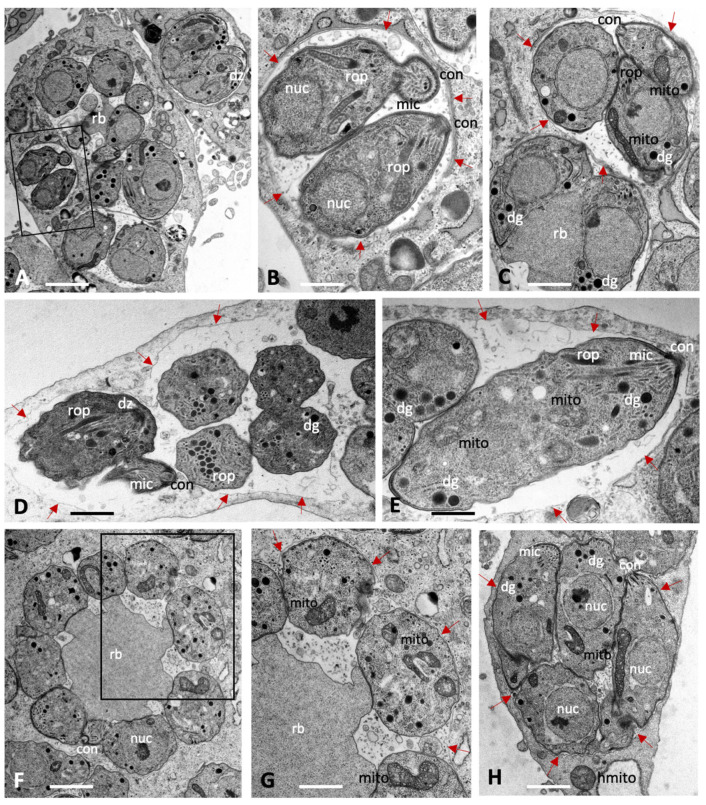
Tachyzoites of *Toxoplasma gondii* (**A**–**C**), *Neospora caninum* (**D**,**E**) and *Besnoitia besnoiti* (**F**–**H**) maintained in human foreskin fibroblasts for 48 h in the absence of compound treatment. The boxed areas in (**A**) and (**F**) are enlarged in (**B**) and (**G**), respectively. Tachyzoites undergo intracellular proliferation in a parasitophorous vacuole, which is delineated by a parasitophorous vacuole membrane (marked with red arrows); nuc = nucleus, rop = rhoptries, mic = micronemes, dg = dense granules, mito = mitochondrion, con = conoid, rb = residual body, dz = newly formed daughter zoites. Bars in (**A**) = 2.3 µm; (**B**) = 0.75 µm; (**C**) = 0.85 µm; (**D**) = 1.1 µm; (**E**) = 0.75 µm, (**F**) = 1.25 µm; (**G**) = 0.75 µm; (**H**) = 1.1 µm.

Figure 1 displays electron micrographs of *T. gondii*, *B. besnoiti* and *N. caninum* tachyzoites grown in HFF monolayers in the absence of BKIs. The three species exhibited a largely similar ultrastructure. Tachyzoites reside and proliferate within a parasitophorous vacuole that is delineated by a parasitophorous vacuole membrane (indicated by red arrows in Figure 1). The parasites displayed the typical apicomplexan features including an apical complex with the conoid, and secretory organelles such as rhoptries, micronemes and dense granules. Depending on the section plane, larger or smaller portions of the single mitochondrion were visible, including the mitochondrial matrix with cristae of varying electron density. Tachyzoites divided by endodyogeny with two daughter zoites being formed that represented clearly separate entities. Frequently, daughter tachyzoites were seen still attached to a residual body.

**Figure 2:**
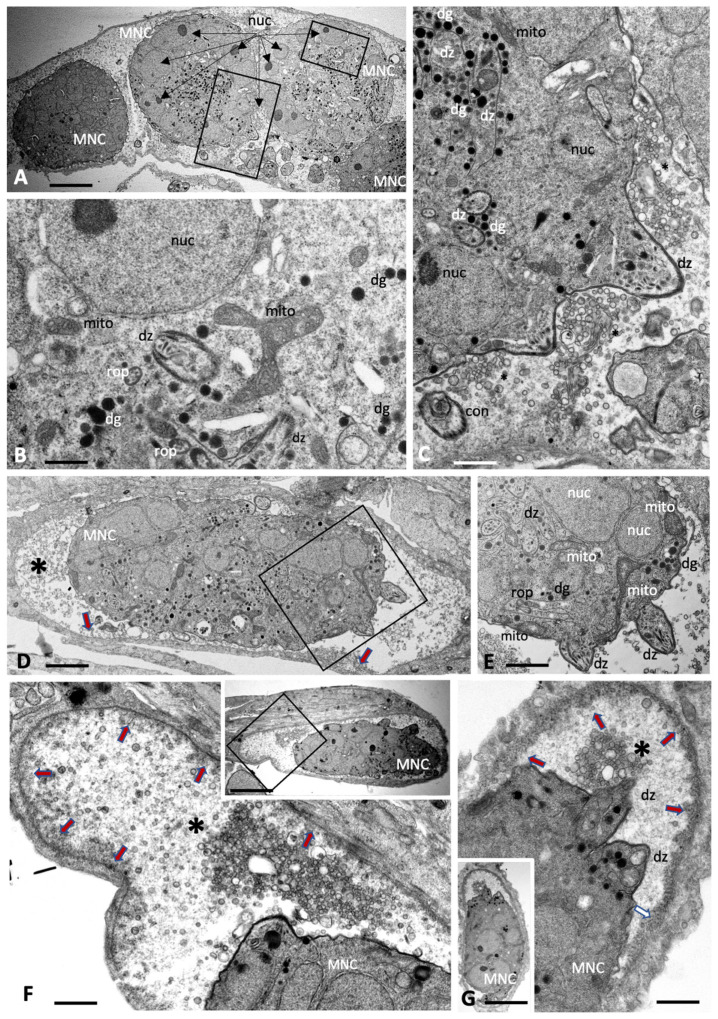
Baryzoites of *T. gondii* grown in human foreskin fibroblast cultures treated with BKI-1708 over 4 (**A**–**C**) and 6 days (**D**–**G**). The boxed areas in (**A**) are shown at higher magnification in (**B**,**C**); the one in (**D**) is magnified in (**E**). Lower magnification views of (**F**,**G**) are shown in the corresponding inserts. MNC = multinucleated complex, nuc = nucleus, rop = rhoptries, dg = dense granules, mito = mitochondrion, dz = apical parts of daughter zoites, * indicates secretory products in the parasitophorous vacuole. The red arrows in (**D**,**F**,**G**) point towards the developing cyst wall. Bars in (**A**) = 3.2 µm; (**B**) = 0.5 µm; (**C**) = 0.75 µm; (**D**) = 3.2 µm; (**E**) = 1.7 µm; (**F**) = 1.2 µm; (**F**) insert = 6.7 µm; (**G**) = 1.5 µm; (**G**) insert = 6.7 µm.

Treatment with BKI-1708 of *Toxoplasma gondii* ME49 cultured in HFFs led to the transformation of intracellular tachyzoites into schizont-like and thus multinucleated baryzoites, while single tachyzoites were not detected (Figure 2). *T. gondii* baryzoites formed after 4 days of treatment are shown in Figure 2A–C, and baryzoites formed after 6 days of BKI-1708 treatment are shown in Figure 2D–G. Baryzoites contained numerous nuclei that form clusters either in the periphery or to one side of the cytoplasm, depending on the section plane. Small apical complexes of daughter zoites were frequently seen protruding at the periphery or within the cytoplasmic areas, which also contain the typical organelles reminiscent for apicomplexans, including dense granules, rhoptries, as well as portions of a structurally intact mitochondrion including an electron-dense matrix and cristae. However, these newly formed zoites lacked the typical triple layered membrane of tachyzoites. Instead, this membrane covered the entire baryzoite surface. *T. gondii* baryzoites were seen to release large amounts of secretory vesicles and lamellar membranous components into the matrix of the parasitophorous vacuole. At 6 days of treatment it appeared that at least some of this material formed electron dense deposits at the periphery of the vacuole (Figure 2F,G). During this remarkable drug-induced transformation, however, based on ultrastructural appearance, parasites were largely viable.

**Figure 3:**
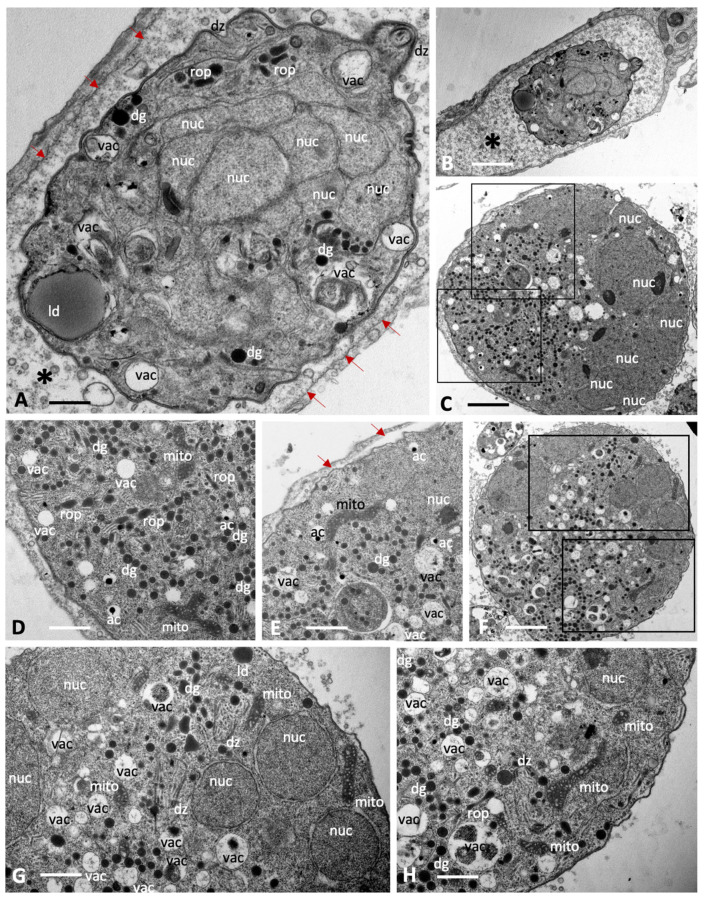
Baryzoites of *N. caninum* grown in human foreskin fibroblast cultures treated with BKI-1708 over 4 (**A**–**C**) and 6 days (**D**–**H**). The MNC in (**A**) is shown enlarged in (**B**), the * marks the interior matrix of the parasitophorous vacuole, red arrows point towards the parasitophorous vacuole membrane. Higher magnification views of the boxed areas in (**C**) are depicted in (**D**,**E**). Selected areas in (**F**) are enlarged in (**G**,**H**); nuc = nucleus, dg = dense granules, rop = rhoptries, vac = cytoplasmic vacuoles, ld = lipid droplets, mito = mitochondrion, ac = acidocalcisome, dz = apical part of a daughter zoite. Bars in (**A**) = 2.3 µm; (**B**) = 0.65 µm (**C**) = 1.6 µm; (**D**) = 0.8 µm; (**E**) = 0.9 µm; (**F**) = 1.6 µm; (**G**) = 0.8 µm; (**H**) = 0.8 µm.

Baryzoites formed upon exposure of *N. caninum* to BKI-1708 (Figure 3) exhibited largely similar characteristics and also remained within their host cell. The matrix of the parasitophorous vacuole was composed of rather evenly distributed vesicular and granular components, but an electron-dense cyst wall-like structure was clearly missing. As in *T. gondii*, nuclei were clustered together and separated from other cytoplasmic compartments of newly formed daughter zoites (Figure 3C,F,G,H), and mitochondria also remained structurally intact (Figure 3D,E,G,H), indicating that there is no impairment of viability. Compared to T. gondii baryzoites, however, *N. caninum* baryzoites contained an increased number of organelles that resembled dense granules (dg) exhibiting variable diameters (150–400 nm). Occasionally lipid droplets (ld) were seen (Figure 3A,G). In addition, an increased number of vacuolar inclusions of varying sizes were observed that either appeared empty or contained undefined granular or filamentous material or electron dense deposits, the nature of which is unknown (Figure 3C–H).

**Figure 4:**
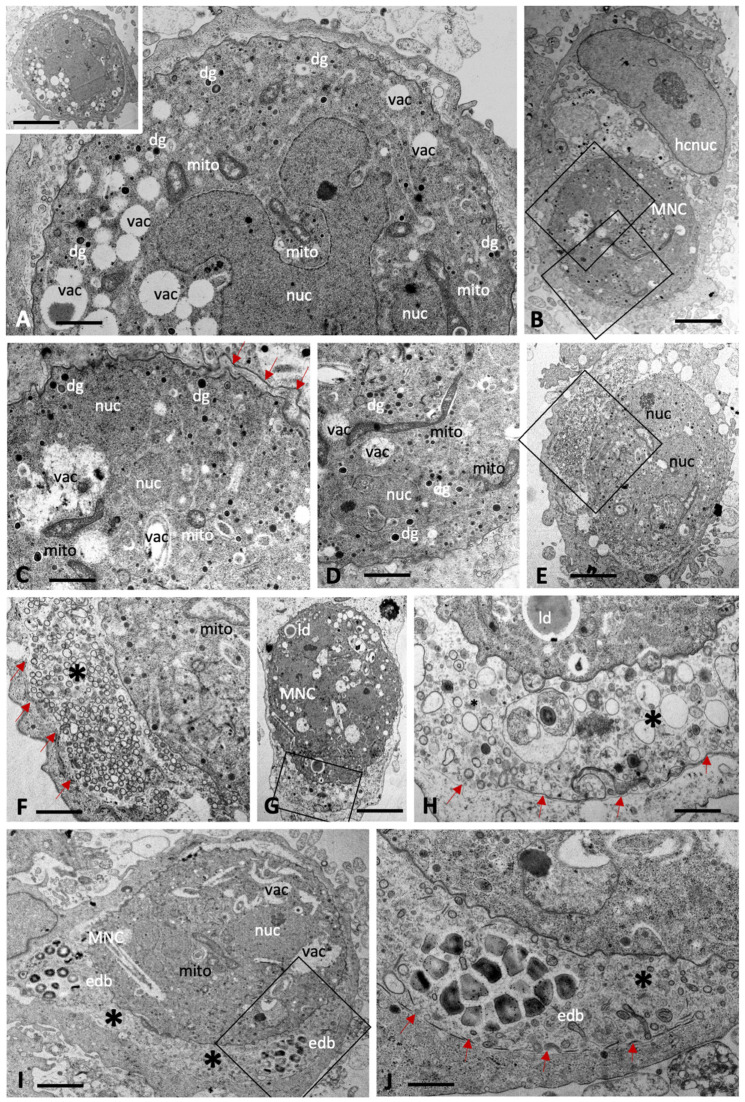
Baryzoites of *B. besnoiti* grown in human foreskin fibroblast cultures treated with BKI-1708 over 4 (**A**–**E**) and 6 days (**F**–**J**). The insert in (**A**) shows a lower magnification view of the respective MNC, and the two boxed areas in (**B**) are enlarged in (**C**,**D**). (**F**) shows an enlarged view of the boxed area in (**E**), and the framed areas in (**G**,**I**) are shown at higher magnification view in (**H**,**I**). Red arrows point towards the parasitophorous vacuole membrane. MNC = multinucleated complexes, nuc = nucleus, dg = dense granules, hcnuc = host cell nucleus, vac = cytoplasmic vacuoles, edb = electron dense bodies, mito = mitochondrion, ld = lipid droplet. * = matrix of the parasitophorous vacuole, arrows point at parasitophorous vacuole membrane. Bars in (**A**) = 0.8 µm; (**A**) insert = 4.2 µm; (**B**) = 2 µm; (**C**) = 0.7 µm; (**D**) = 1.2 µm; (**E**) = 2 µm; (**F**) = 0.7 µm, (**G**) = 3.6 µm, (**H**) = 0.7 µm, (**I**) = 2.4 µm; (**J**) = 0.8 µm.

*B. besnoiti* baryzoites exhibited similar features, with nuclei and cytoplasmic components largely compartmentalized (Figure 4). However, the degree of vacuolization appeared higher compared to *T. gondii* and *N. caninum*, including also irregularly shaped vacuoles. In many baryzoites, the central portion of the mitochondrial matrix appeared slightly distorted but more electron dense in the periphery (Figure 4F). Generally, the typical apicomplexan secretory organelles such as micronemes, rhoptries and dense granules were less well preserved. Numerous dense granules were found in the cytoplasm, albeit of small size (200 nm or below). In *B. besnoiti,* the matrix of the parasitophorous vacuole (indicated with an asterisk) was filled with small secreted vesicles (Figure 4E,F), as well as also larger vesiculated structures (Figure 4G,H), and additionally clusters of electron dense bodies (Figure 4I,J) of unknown composition.

### 2.2. BKI-1708 Does Not Act Parasiticidal and Removal of BKI-1708 Leads to Reversion to Tachyzoites

**Figure 5:**
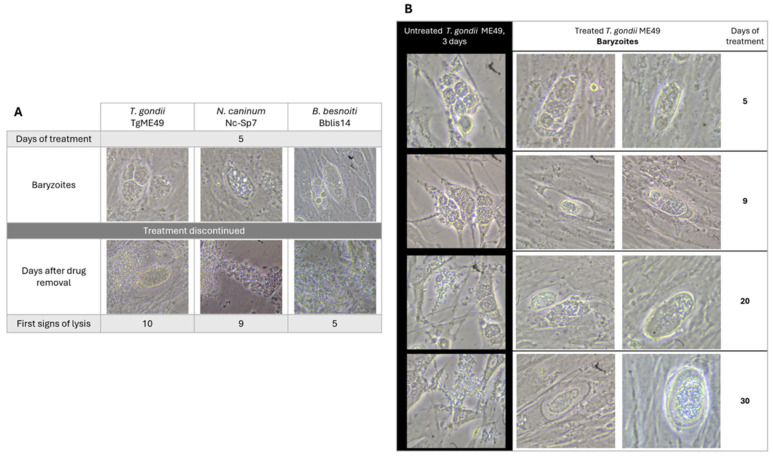
Reversion and long treatment assays. (**A**) Tachyzoites were treated with BKI-1708 at 2.5 μM from 1 h post-infection for 5 consecutive days, leading to the formation of baryzoites. On day 5, drug pressure was released, and cultures were monitored daily by conventional light microscopy until lysis plaques are detected. Regrowth of parasites was noted in all cases. (**B**) ME49 tachyzoites were treated for a total period of 30 days and monitored by conventional light microscopy. Intact baryzoites were observed even after 30 days of continuous treatment.

Infected cultures were treated with 2.5 µM BKI-1708 for 5 days, followed by culture in the absence of drug treatment and daily microscopical inspection. All the three species were able to recover from treatment and reverted from baryzoites into the infective tachyzoite stage, but they did so at different rates (Figure 5A).

*B. besnoiti* baryzoites resumed proliferation more rapidly, with baryzoite-to-tachyzoite reversion observed after 5 days of drug removal. *T. gondii* and *N. caninum* baryzoites required 10 and 9 days, respectively, to revert into infective tachyzoites. In addition, prolonged treatment of *T. gondii*-infected cultures for a period of 30 days did not clear the infection or lead to disintegration of the complexes (Figure 5B). Baryzoites remained intact and morphologically similar to those observed after shorter treatment periods.

### 2.3. Upon Treatment with BKI-1708, T. gondii Baryzoites Upregulate Bradyzoite-Specific Markers and Downregulate Tachyzoite Antigens

**Figure 6:**
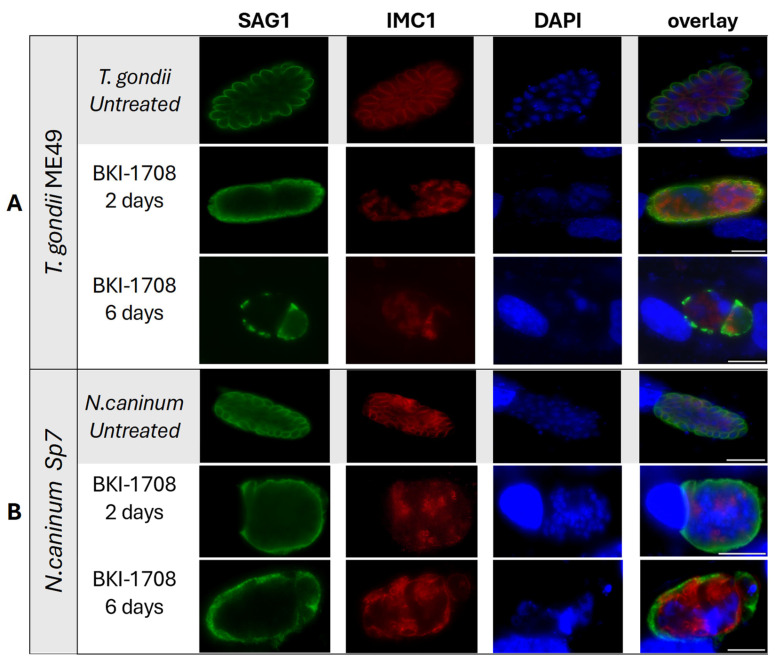
Immunofluorescence staining of *T. gondii* (**A**) and *N. caninum* (**B**) untreated (control) and treated tachyzoites (baryzoites) after 2 and 6 days. Green labels the major tachyzoite surface antigen (SAG1). Red delimitates individual zoites, labeling the inner membrane complex (IMC1). DNA of nuclei is blue, stained by DAPI. Scale bars = 10 μm.

As shown by immunofluorescence in Figure 6, the typical untreated apicomplexan tachyzoites of *T. gondii* and *N. caninum* were labeled with antibodies directed against TgSAG1 and NcSAG1, respectively, and antibodies directed against IMC. In *T. gondii* treated with BKI-1708. TgSAG1-staining no longer defined the contours of individual tachyzoites but was found on the surface the developing baryzoites, with diminished staining intensity after 6 days of treatment (Figure 6A). A similar phenotype was observed in *N. caninum* baryzoites, where BKI-1708 treatment also resulted in altered NcSAG1 localization, accumulation of nuclei and IMC1-stained zoites (Figure 6B). Overall, these results mirrored earlier reports on the effects of BKI-1294 in *N. caninum* [19].

**Figure 7:**
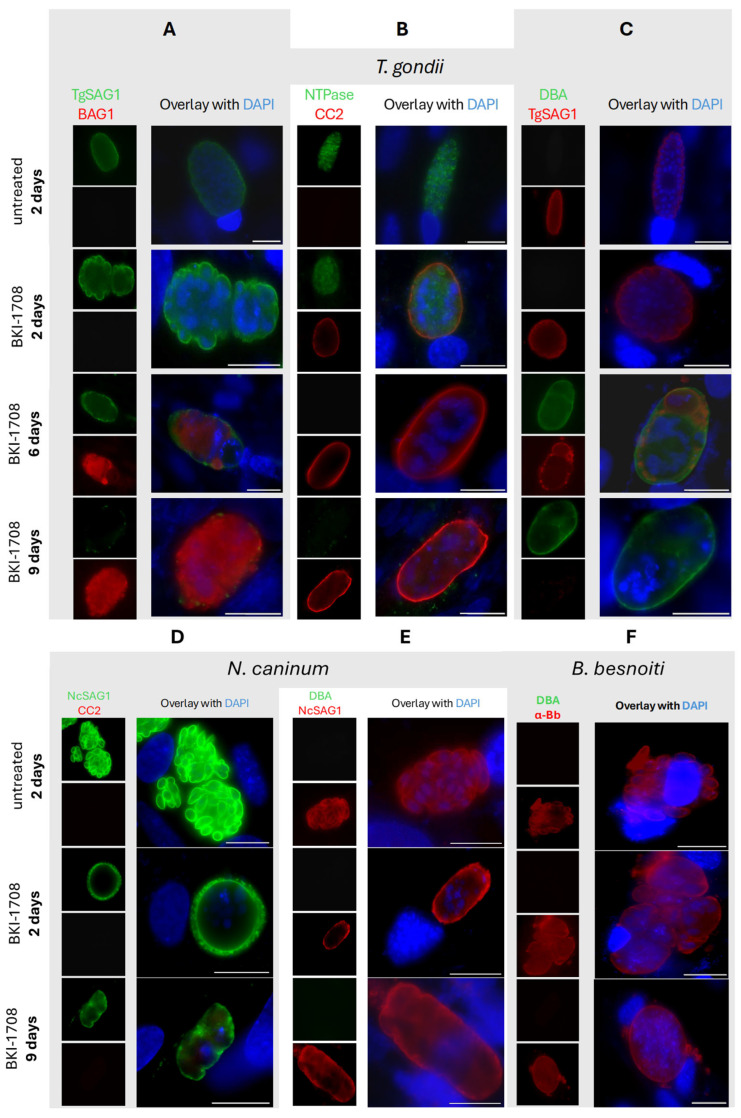
Immunofluorescence of *T. gondii* (**A**–**C**), *N. caninum* (**D**,**E**) and *B. besnoiti* (**F**) under BKI-1708 treatment. (**A**) Major surface antigen SAG1 is stained in green. Red labels bradyzoite cytoplasmic proteins (BAG1). (**B**) Nucleoside-triphosphatase (NTPase) enzyme is labeled green. α-CC2 staining is red. (**C**) Dolichos biflorus agglutinin (DBA) is stained with green. Major surface antigen SAG1 is stained in red. (**D**) NcSAG1 major antigen stained in green, CC2 labeled in red. (**E**) NcSAG1 stained in red, DBA is labeled in green. Anti-*Besnoitia* spp. staining is red (**F**) and DBA is labeled green. Nuclei in all panels were stained with DAPI (blue). Scale bars = 10 μm.

Prolonged exposure to BKI-1708 for 9 days resulted in the disappearance of detectable TgSAG1 staining on the *T. gondii* baryzoites (Figure 7A,C). From 6 days of treatment onwards, *T. gondii* baryzoites expressed the bradyzoite marker TgBAG1) (Figure 7A). In addition, *T. gondii* baryzoites switched on the expression of cyst wall-associated markers recognized by mAb CC2 and DBA (Figure 7B,C). However, in *N. caninum* baryzoites, NcSAG1 expression on the baryzoite surface remained unaltered even after 9 days of BKI-1708 exposure, while no labeling was detected using the cyst wall markers mAbCC2 and DBA (Figure 7D,E). Antibodies directed against TgBAG1 also did not react with *N. caninum* baryzoites and none of the fluorescent markers labeled *B. besnoiti* baryzoites (Figure 7F).

### 2.4. Comparative Proteomics Study of BKI-1708 Treated Baryzoite Cultures Reveals Differentially Expressed Proteins in T. gondi, N. caninum and B. besnoiti

**Figure 8:**
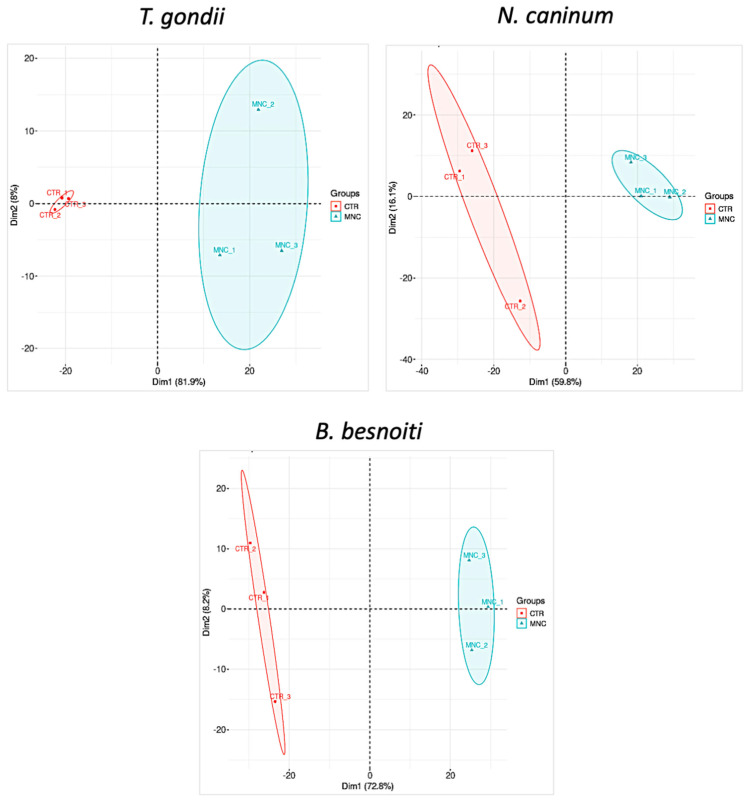
Proteomic profiling of multinucleated complexes (baryzoites) in *Toxoplasma gondii*, *Neospora caninum*, and *Besnoitia besnoiti*. Principal component analysis (PCA) of log_2_-transformed SN intensities from proteome datasets of untreated tachyzoites (CTR, red color) and treated parasites forming multinucleated complexes (MNC (blue color)) for *T. gondii* ME49, *N. caninum* Nc-Sp7, and *B. besnoiti* Bblis14.

**Figure 9:**
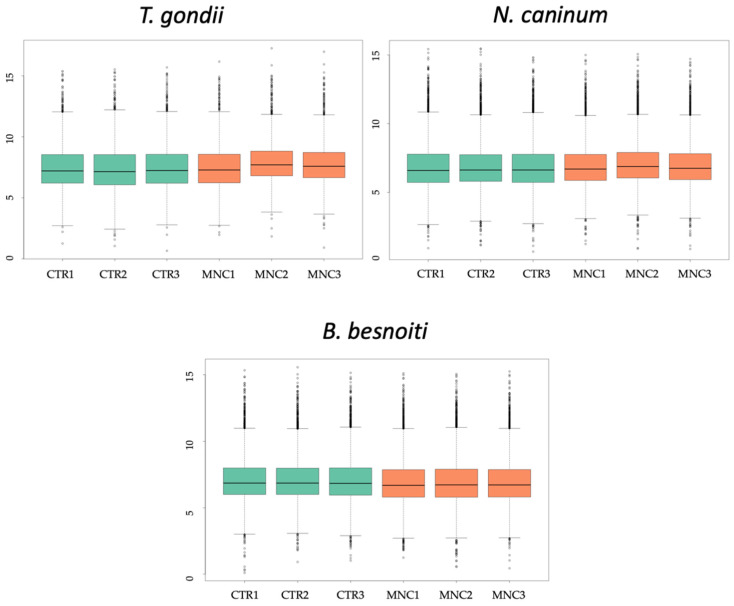
Box plots of log_2_-transformed leading SN intensities of the same proteome datasets comparing untreated tachyzoites (CTR) and MNC for the three species. MNC refers to multinucleated complexes or baryzoites.

*T. gondii*-, *N. caninum*- and *B. besnoiti*-infected HFF were treated with BKI-1708 for 5 days and were subjected to comparative proteomic analyses, thus revealing the presence of differentially expressed proteins in baryzoites compared to untreated tachyzoites. Overall, 2670 proteins were identified in cultures infected with *T. gondii* baryzoites, 1461 in *N. caninum*, and 2755 proteins were identified in *B. besnoiti* baryzoite cultures (Table 1). Unbiased analysis of the dataset via principal component analysis (PCA) of the log2-transformed Spectronaut (SN) intensities of the samples demonstrated that the proteomes of tachyzoites and BKI-1708-induced baryzoites formed non-overlapping clusters separated by both principal components (Figure 8). In addition, SN protein intensity distributions were almost equal for all samples (Figure 9). The entire proteomic datasets for *T. gondii*, *N. caninum* and *B. besnoiti* are presented in Supplementary Tables S1, S2 and S3, respectively, and the respective volcano plots are shown in Supplementary Figure S1.

The basic hallmarks of the three proteomes and respective differentially expressed (DE) proteins are depicted in Table 1. For all three species, the number of baryzoite proteins with downregulated expression levels compared to tachyzoites was higher than the number of proteins with upregulated expression levels. *B. besnoiti* baryzoite cultures exhibited the strongest downregulatory response to treatment, with 14.1% of all proteins expressed at lower abundance, compared to 10.9% of *T. gondii* and 7.8% of *N. caninum* proteins. Conversely, upon BKI-1708 treatment, the largest proportion of proteins was upregulated in *T. gondii* baryzoites (5.7%) compared to *B. besnoiti* and *N. caninum* baryzoites (1.4 and 1%, respectively). A total of sixteen orthologs were commonly found in all three species at lower abundance in baryzoite cultures, while three were consistently found to be expressed at higher levels compared to untreated tachyzoites in all three species (Table 2).

**Figure 10:**
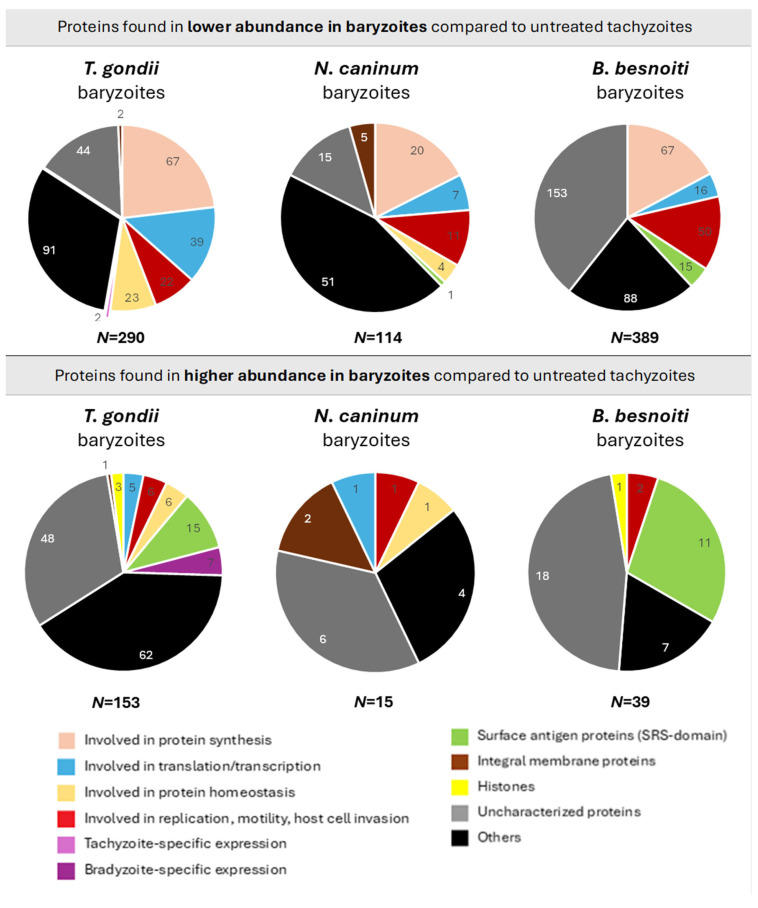
Differentially regulated proteins detected in baryzoites compared to untreated tachyzoites of *Toxoplasma gondii*, *Neospora caninum*, and *Besnoitia besnoiti*, categorized by their biological functions. “Uncharacterized proteins” include hypothetical proteins, and “Others” refer to detected proteins with unknown functions or those not classified within the defined categories. Functional categories were assigned to each protein according to the information retrieved from ToxoDB (https://toxodb.org; release 68, 7 May 2024; accessed 1 September 2025). The number of proteins differentially regulated in a given pathway are indicated with small numbers, and *N* = total number of represented proteins. The relative abundances of all proteins in treated and non-treated parasites can be found in Table S1 (DE proteins in *T. gondii*), Table S2 (DE proteins in *N. caninum*) and Table S3 (DE proteins in *B. besnoiti*).

The DE proteins of all three species were grouped into categories according to their putative functions (Figure 10). A description of each category is provided in Supplementary Table S4. However, across all three species, a significant part of the DE baryzoite proteins were uncharacterized proteins, meaning hypothetical proteins predicted on genomic data. This shows that large portions of these genomes still need to be annotated (Figure 10).

Nevertheless, based on putative functional activity, a consistent pattern of protein downregulation was observed in baryzoites of all three species, with the same general groups of proteins exhibiting decreased levels, despite some parasite-specific differences. Following treatment, proteins involved in translation, transcription, replication, motility, and host cell invasion were less abundant in all parasites, with ribosomal proteins being most prominently affected: of the sixteen proteins that were found to be commonly expressed at lower abundance, only calmodulin and profilin did not represent ribosomal constituents, suggesting an impact on protein synthesis (Table 2).

Several SRS-domain surface antigen proteins were expressed at reduced abundance in *B. besnoiti*, while only one was similarly affected in *N. caninum*, and none in *T. gondii*. The list of SRS-domain surface antigen proteins differentially regulated in baryzoites exposure is given in Table 3.

**Figure 11:**
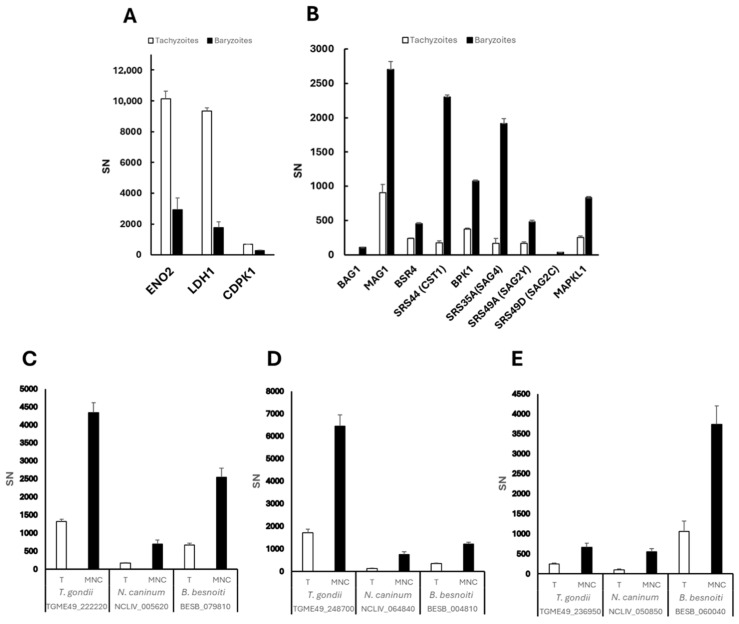
DE proteins found at lower (**A**) and higher (**B**–**E**) abundance in BKI-1708-treated *T. gondii* compared to untreated cultures. DE proteins with significantly higher levels in treated cell cultures (baryzoites) compared to untreated cultures (tachyzoites) in *T. gondii*, *N. caninum*, and *B. besnoiti* include: (**C**) alveolin-domain intermediate filament IMC7; (**D**) alveolin-domain intermediate filament IMC12; and (**E**) a hypothetical protein. DE proteins were identified as described in the Materials and Methods section. Spectronaut intensities (SN) are given as mean ± standard deviation for three replicates. T—tachyzoites; MNC—multinucleated complexes or baryzoites.

Reduced expression in *T. gondii* baryzoites was also noted for the tachyzoite markers enolase 2 (ENO2; TGME49_268850), lactate dehydrogenase 1 (LDH1; TGME49_232350), and calcium dependent protein kinase 1 (CDPK1; TGME49_301440), which is one of the BKI-targets identified to date (Figure 11A). These markers were not affected in *N. caninum* or *B. besnoiti*. CDPK1 orthologues in *N. caninum* (BN1204_044860) and *B. besnoiti* (BESB_015950) were detected in lower abundance in baryzoites compared to untreated parasites, although the variation was not statistically significant.

Among the 153, 15 and 39 proteins that were detected at higher abundance in baryzoites of *T. gondii*, *N. caninum* and *B. besnoiti*, respectively, only three were upregulated in all three species. These include orthologues of the two alveolin domain containing intermediate filament proteins IMC12 (NCLIV_064840, BESB_004810, TGME49_248700) and IMC7 (NCLIV_005620, BESB_079810, TGME49_222220), and a hypothetical protein (NCLIV_050850, BESB_060040, TGME49_236950) (Figure 11C–E).

The 10 most highly abundant proteins of the 153 with increased expression levels in *T. gondii* baryzoites are shown in Table 4. In response to treatment, *Toxoplasma gondii* exhibits significant upregulation of specific histone variants, notably histone H3.3 (TGME49_218260), H2A.Z (TGME49_300200) and CENH3 (TGME49_225410). These variants play important roles in chromatin dynamics and gene regulation: H3.3 is associated with active transcription and chromatin remodeling; H2A.Z is implicated in transcriptional regulation and chromatin stability; and CENH3, a centromeric histone variant, is crucial for proper chromosome segregation during cell division [21]. The increased levels of these histone variants and alveolin-domain intermediate filaments IMC7 and IMC12 in baryzoites suggest that BKI-1708 treatment induces specific coordinated changes in chromatin and cytoskeletal remodeling events, consistent with the observed alterations in cell cycle dynamics (Figure 2, Figure 3, Figure 4, Figure 5 and Figure 6).

Within the calmodulin-like protein family, the essential light chain ELC2 (TGME49_305050) (ortholog of *N. caninum* NCLIV_001300 and *B. besnoiti* BESB_025690) was downregulated across species (Table 2), whereas calmodulin (TGME49_249240) was specifically upregulated in *T. gondii* (Table 4), possibly suggesting recruitment of an alternative calmodulin to support calcium signaling and stress adaptation [22]. Among other proteins with increased abundance were bradyzoite markers such as TgBAG1 (TGME49_259020), the matrix antigen 1 TgMAG1 (TGME49_270240), as well as bradyzoite-specific SRS-domain proteins such as BSR4 (TGME49_320180), SRS44 (TGME49_264660), SRS35A (TGME49_280570), SRS49A (TGME49_207130), SRS49D (TGME49_207160), and the pseudokinase BPK1 (TGME49_253330), which mediates infectivity of *T. gondii* tissue cysts (Figure 11B). Moreover, BKI-1708 treatment resulted in increased expression of the secondary BKI-target MAPKL1 (TGME49_312570) (Figure 11B). The corresponding orthologs of TGME49_312570 in *N*. *caninum* and *B*. *besnoiti* are NCLIV_056080 and BESB_073700, respectively. Comparative analysis of the amino acid sequences of ATP binding sites suggests that they are highly conserved (Supplementary Figure S2). However, in our analysis, we could not detect the MAPKL1 orthologues NCLIV_056080 and BESB_073700 in the *N. caninum* and *B. besnoiti* proteomes, neither in tachyzoites nor in baryzoites. Also, noteworthy, serine palmitoyltransferase SPT2 (TGME49_290970), a key enzyme in ceramide biosynthesis, was detected in *T. gondii* baryzoites but not in untreated tachyzoites [23].

No bradyzoite-specific markers were found to be expressed at higher levels in *N. caninum* or *B. besnoiti* baryzoites. In addition, two uncharacterized proteins (TGME49_226570 and TGME49_248360) were found at higher levels in treated *T. gondii* and *N. caninum*, but not in *B. besnoiti*. Their respective orthologues in *N. caninum* are NCLIV_046410 and NCLIV_064330.

## 3. Discussion

Here, we present a comparative study of *T. gondii*, *N. caninum*, and *B. besnoiti* baryzoites. Baryzoites are multinucleated complexes that are formed upon exposure of these parasites to the bumped kinase inhibitor BKI-1708, a compound that had earlier shown promising efficacy against *N. caninum* and *T. gondii* infection in vitro and in pregnant mouse models [20]. We thus report on the similarities and differences between the three species by focusing on the impact of BKI-1708, a BKI-lead compound, on ultrastructure, parasitostatic activity, the expression and localization of bradyzoite and tachyzoite markers, and proteomics.

Comparative TEM analysis showed that in vitro treatments of 2.5 μM BKI-1708-induced baryzoite formation in all three species. Inspection by TEM revealed many ultrastructural similarities between the three species. Overall, baryzoites represented a complex of newly formed zoites that failed to complete cytokinesis. Consequently, disjunction and the formation of infective tachyzoites were inhibited. Parasites remained trapped within the host cell, while DNA-replication and nuclear division appeared to continue. Thus, nascent apical complexes, conoids, and respective secretory organelles commonly found in apicomplexans, including rhoptries, dense granules and micronemes, were visible within the baryzoite matrix, while nuclei of newly formed zoites were clustered together. In several instances, newly formed anterior ends of the parasites pointed outwards to the periphery. These cytoplasmic elements were most clearly discernible in *T. gondii* baryzoites. BKI-1708-induced baryzoites were structurally similar to those previously observed following treatments of *T. gondii* and *N. caninum* with the PP compound BKI-1294 [19] and the AC compound BKI-1748 [14], and to those of *B. besnoiti* treated with a series of other PP and AC BKIs [24].

Despite considerable changes induced by BKI-1708, baryzoites appeared to be viable and metabolically active, as indicated by the presence of mitochondria with a more or less intact mitochondrial matrix and cristae. Treatment resulted in a complete absence of plaque formation, indicating that tachyzoites failed to successfully complete lytic replication. Viability of baryzoites was confirmed by regrowth experiments. Under longer-term continuous BKI-1708 drug pressure over 30 days, *T. gondii* baryzoites remained morphologically intact and treatments did not result in clearance of infection. It is unclear, however, whether these forms were still metabolically active, and regrowth after drug removal was not assessed. Earlier studies had reported that viable parasites resumed proliferation upon drug removal after 20 days of treatment with 5 μM BKI-1294 [4], also suggesting ongoing metabolic activity for extended periods of time.

TEM revealed several distinguishing structural features in baryzoites of each organism. On one hand, BKI-1708 treatments resulted in a higher number of cytoplasmic vacuoles and lipid droplets in the *Neospora* and *Besnoitia* baryzoite cytoplasm compared to *T. gondii*. Cytoplasmic vacuolization and the formation of lipid droplets could be indicators of metabolic stress [4] and suggested that BKI-1708 treatment had a more significant metabolic impact on *Neospora* and *Besnoitia* compared to *T. gondii*. Among the proteins identified in higher abundance in *N. caninum* and *B. besnoiti* baryzoites, several have potential roles in intermediary metabolism and apoptosis. In *N. caninum*, these include a metallophosphoesterase (NCLIV_009030) and a 2OG-Fe(II) oxygenase (NCLIV_042790), enzymes implicated in various metabolic pathways in other organisms [25,26]. Additionally, reflecting on the roles of similar proteases in *T. gondii*, *P. falciparum* and other eukaryotes [27,28], the cathepsin B-like protease (NCLIV_069550), found at higher levels in *N. caninum* baryzoites, likely contributes to lysosomal protein turnover, autophagy, and apoptosis regulation [29,30]. Also in *B. besnoiti*, five proteins found at higher abundance in treated parasites could be linked to intermediate metabolism. Such changes in protein abundance could potentially explain/reflect the severe metabolic stress phenotype observed specifically in BKI-1708-treated *Neospora* and *Besnoitia*.

A second difference concerned the dense granules, which were much more numerous and smaller in size in *Neospora* and *Besnoitia* baryzoites. However, no alterations were noted in the expression levels of dense granule proteins in these parasites. The most notable difference, however, concerned the presence of an electron-dense cyst wall-like structure that appeared at the periphery of the parasitophorous vacuole of *T. gondii* baryzoites after 6 days of BKI-1708 treatment. No such structures were visible after 4 days of treatment, suggesting that the material accumulated at the periphery in a time-dependent manner. In contrast, *N. caninum* and *B. besnoiti* baryzoites did not form a cyst wall. The build-up of a cyst wall by *T. gondii* baryzoites was confirmed by fluorescent labeling with the tissue cyst markers DBA and mAbCC. The lectin DBA binds to the cyst wall glycoprotein 1 (CST1), a 116 kDa glycoprotein that plays a crucial role in maintaining cyst wall integrity and is essential for bradyzoite persistence within host tissues [31]. The mAbCC2 recognizes a 40 kDa dense granule antigen in tachyzoites and recognizes a 115 kDa cyst wall protein that is upregulated during *T. gondii* bradyzoite differentiation [32]. In *N. caninum*-infected host cells treated with sodium nitroprusside, both DBA and mAbCC2 had also labeled the walls of in vitro-generated tissue cysts containing bradyzoites [33]. Furthermore, increased staining with mAbCC2 had also been observed in both *T. gondii* and *N. caninum* baryzoites induced by BKI-1294 treatment, with preferential peripheral staining on *T. gondii* and cytoplasmic staining in *N. caninum* baryzoites [4]. However, upon treatment with BKI-1708, these antibodies failed to react with *N. caninum* or *B. besnoiti* baryzoites. Another bradyzoite marker, the bradyzoite antigen 1 (BAG1), a heat shock protein 30 (HSP30)-related protein associated with tachyzoite-to-bradyzoite conversion [33,34,35], was found to be expressed in *T. gondii*, but not in *N. caninum* or *B. besnoiti* baryzoites. We also performed immunofluorescence staining with antibodies against the major tachyzoite surface antigen 1 (SAG1) [36,37]. TgSAG1 labeling on the baryzoite surface in *T. gondii* got progressively weaker during BKI-1708 treatment and was completely diminished after 9 days. In contrast, in *N. caninum* baryzoites undergoing the same BKI pressure, the NcSAG1 signal persisted and remained visible even after 9 days of BKI exposure. Although orthologs of SAG1 and BAG1 have been identified in *B. besnoiti* [3,38], antibodies reacted neither with *B. besnoiti* tachyzoites nor baryzoites. Overall, this indicated that the BKI-1708-induced baryzoites of *T. gondii* were more closely related to the tissue cysts generated by bradyzoites than their counterparts in *N. caninum* and *B. besnoiti*, which is in line with the results on the respective proteomes.

*T. gondii-, N. caninum-* and *B. besnoiti*-infected HFF were treated with 2.5 μM of BKI-1708 for 5 days to induce the formation of baryzoites. Protein abundances were assessed by label-free quantitative proteomics using the Spectronaut algorithm [39] and analyzed in comparison to untreated tachyzoites. Proteins showing significant abundance changes compared to untreated samples were defined by a log_2_ fold change ≥1 and *p*-value < 0. The total number of proteins detected was highest in *B. besnoiti* (2755), followed by *T. gondii* (2670), and lowest in *N. caninum* (1461). A higher proportion of downregulated proteins was found in all treated strains, particularly in *B. besnoiti* (14.1%) while the proportion of upregulated proteins in response to BKI-1708 treatment was rather low across all three species, ranging from 1 to 5%. In an analogy to the ultrastructural changes in *B. besnoiti* baryzoites that were more pronounced than those observed in *T. gondii* or *N. caninum,* proteomic profiling revealed that *B. besnoiti* exhibited (i) the strongest downregulatory response and (ii) the highest upregulation of expressed proteins potentially associated with metabolism and apoptosis under BKI-1708 treatment, suggesting a more pronounced shutdown of cellular functions compared to the other species. Overall, 19 orthologs showed consistent abundance changes across all three species, of which 16 were found at lower, and 3 at higher abundance.

Grouping DE proteins into functional categories illustrated that proteins involved in the assembly and function of the ribosomes were most notably affected. Despite some parasite-specific differences, proteins involved in translation, transcription, replication, motility, and host cell invasion also exhibited lower abundances in all parasites, likely reflecting a common metabolic shutdown response associated with BKI-1708 treatments. Notable differences were detected with respect to the expression of SAG1-related sequence (SRS) proteins, with several SRS proteins less abundant in *B. besnoiti* baryzoites, only one SRS protein with decreased expression in *N. caninum* baryzoites, and no SRS domain-containing protein being downregulated in *T. gondii* baryzoites. This was not in agreement with the immunofluorescence results suggesting a progressive decrease in expression with time, although most effectively after 9 days of treatment. Thus, the discrepancy can be explained by the earlier timepoint of sample collection for proteomics. However, a substantial portion of proteins upregulated in response to BKI-1708 treatments were SRS proteins, namely 10% in *T. gondii* and 28% in *B. besnoiti* baryzoites. In contrast, no increased expression of SRS proteins was noted for the *N. caninum* baryzoite proteome. Earlier findings on the effects of BKI-1294 treatments of *N. caninum* had shown a different picture: of the 12 proteins exhibiting higher abundance in baryzoites, 6 were SRS domain containing proteins [19]. Nonetheless, the bradyzoite-specific protein bradyzoite surface receptor 4 (BSR4) was detected in *N. caninum* baryzoites and not detected in untreated parasites, though it was not considered significantly differentially expressed according to the parameters of the applied DE test. Although variations in experimental settings between studies (e.g., incubation times, BKIs with different scaffolds, algorithms used for proteomics) prevent direct comparisons, other similarities in parasite responses were observed, such as reduced levels of housekeeping and intermediate metabolism proteins in BKI-1294-induced *N. caninum* baryzoites that align with observations in BKI-1708-induced baryzoites in *T. gondii*, *N. caninum* and *B. besnoiti* [19].

While immunofluorescence already indicated that *T. gondii* baryzoites, in contrast to *N. caninum* and *B. besnoiti,* move towards a bradyzoite-like expression pattern, this was confirmed on the proteomic level: tachyzoite markers such as enolase 2 (ENO2) and lactate dehydrogenase 1 (LDH1) were downregulated. The same was observed for the BKI target CDPKCDPK1, which is essential for signaling leading to microneme secretion and thus host cell invasion; thus downregulation of its expression in baryzoites would have no impact on viability. In this study, no changes in expression levels of CDPK1 orthologues were found in *N. caninum* and *B. besnoiti* baryzoites. However, it is important to note that functional divergence and independent evolution of the orthologs in different species may influence their response levels under specific conditions. Bradyzoite markers such as BAG1, matrix antigen protein 1 (MAG1), and several SRS proteins were expressed at higher levels in *T. gondii* baryzoites. In addition, MAPKL1 (TGME49_312570), more recently identified as an alternative BKI-target in *T. gondii* [17], was more abundantly expressed in baryzoites than in tachyzoites. MAPKL1 is a coccidian-specific kinase that is localized to the pericentrosomal material and is essential for the regulation of centrosome duplication during endodyogeny. The presence of BKI-1708 most likely impacts on the activity of this kinase, which could be compensated for by upregulated expression in baryzoites, thus maintaining continued replication and nuclear division. Comparative sequence analysis for the corresponding orthologs of TGME49_312570, NCLIV_056080 in *N. caninum* and BESB_073700 in *B. besnoiti* suggests that they are highly conserved. This would imply that *N*. *caninum* and *B*. *besnoiti* baryzoites should also have increased expression of NCLIV_056080 and BESB_073700 after BKI-1708 treatment. MAPKL1 orthologues in *B. besnoiti* and *N. caninum*, however, were not detected in the respective proteomes, neither in tachyzoites nor in baryzoites, suggesting that they are expressed at very low levels, or the proteins were not stable under the conditions used. The absence of bradyzoite markers in *N. caninum* baryzoites may be attributed to the sensitivity limitations of the proteomic methods employed [40]. Alternatively, the absence of bradyzoite markers reflect species-specific differences in bradyzoite formation. *N. caninum* bradyzoite formation in vitro is inherently more difficult to achieve, whereas certain *T. gondii* strains can even differentiate spontaneously, and bradyzoite formation is a well-characterized process [29,30].

Among all identified proteins, 19 are commonly differentially regulated across all BKI-1708-induced baryzoites. Of these, 16 proteins were consistently detected at lower levels in baryzoites, 14 of which are ribosomal proteins, thus reflecting a general decrease in protein synthesis and a transition from an actively replicating state to a dormant form.

Two additional commonly downregulated proteins were calmodulin (NCLIV_001300, TGME49_305050, BESB_025690) and profilin (NCLIV_000610, TGME49_293690, BESB_082700). Calmodulin is a small, Ca^2+^-binding protein, on which apicomplexan parasites rely on for essential functions such as protein secretion, motility, host cell invasion and egress [41,42]. Of note, CDPK1, the kinase that is targeted by BKI-1708, contains a C-terminal Ca^2+^ calmodulin domain regulating its activity. In the presence of calcium, this domain undergoes a conformational change, allowing CDPK1 to activate and regulate downstream cellular processes including calcium-triggered microneme secretion [43,44]. Profilin is a small actin-binding protein localized at the apical end of tachyzoites, and is essential for gliding motility, host cell invasion, and active egress from host cells in *Toxoplasma gondii* [45]. Profilin regulates the formation and depolymerization of short actin filaments in the inner membrane of the apical complex, enabling the translocation of myosins and adhesins [46,47]. *T. gondii* profilin is efficiently recognized by the immune system through binding to TLR11 and TLR12 and activates murine dendritic cells and macrophages to release IL-12, which in turn leads to the production of IFN-γ via the MyD88 dependent pathway and induction of Th1 biased immunity. Thus, profilins have been incorporated into vaccine candidates against *T. gondii* [48] and *N. caninum* infection [49].

Furthermore, orthologs of three proteins were detected at higher abundance across all strains. These include the alveolin domain containing intermediate filament protein IMC7 (orthologues: TGME49_222220, NCLIV_005620, BESB_079810), the alveolin domain containing intermediate filament protein IMC12 (orthologues: TGME49_248700, NCLIV_064840, BESB_004810), and an uncharacterized protein (orthologues: TGME49_236950, NCLIV_050850, BESB_060040). The inner membrane complex (IMC) is a double-membrane complex composed of alveolin-family proteins and essential for maintaining cell shape, motility, and host cell invasion, important for parasite stability and intracellular replication [50]. Both IMC7 and IMC12 are essential for the structural integrity of the IMC in *T. gondii*. Their roles are particularly critical during the formation of daughter cells, replication and overall fitness [50]. IMC7 and IMC12 filaments are typically incorporated into the mature cytoskeleton during the G1 phase of the cell cycle, when cytokinesis is complete [51]. The expression of IMC proteins is tightly regulated during the cell cycle [52]. In the context of baryzoites, the accumulation of these IMC-complex filament proteins in baryzoites could be explained by BKI interference with regulatory factors, such as transcription factors or kinases and/or arrest or delay of the G1 phase.

Another notable finding was the consistent detection of high levels of the uncharacterized protein TGME49_236950 (orthologues: NCLIV_050850, BESB_060040) in BKI-1708-induced baryzoites. This hypothetical protein is highly conserved in apicomplexans, yet its function remains unknown, and it shares similarities with redox activators of prokaryotic corrinoid proteins, cobalt-containing cofactors involved in methyl-transfer reactions (reviewed in [53]). In *T. gondii* ME49 artemisinin-resistant strains, TgME49_236950 is downregulated, which could be linked to a gain-of-function with respect to these compounds—either through direct activation of artemisinins or by indirectly repressing proteins that interfere with their metabolic activation [53]. Interestingly, in BKI-1708-induced baryzoites, this protein is consistently found at higher abundance. The increased abundance of TgME49_236950, NCLIV_050850, and BESB_060040 in *T. gondii, N. caninum* and *B. besnoiti* baryzoites, respectively, could reflect a role in stress adaptation and metabolic remodeling during tachyzoite–baryzoite conversion since its similarity to redox activators suggests a potential function in managing oxidative stress or participating in drug metabolism. Alternatively, this upregulation could result from stage-specific expression or compensatory pathway activation triggered by drug-induced signaling changes. Further functional studies would be required to clarify whether these orthologues play an active role in drug response, stage conversion, or broader redox and metabolic processes.

Finally, while this study has identified a number of features of baryzoites across *B. besnoiti*, *T. gondii*, and *N. caninum*, it is important to note that a substantial proportion of the proteomes comprise uncharacterized proteins. These proteins, “hypothetical proteins” are predicted based on open reading frames (ORFs) identified through genomic sequencing but lack experimental validation of their expression or functional characterization. This represents a substantial hurdle for clarification of pathways involved in this parasitostatic response to treatments with BKI. Whether any of the proteins expressed at lower or higher abundance in baryzoites are indeed the ones directly involved in baryzoite formation is not known. Possibly, additional effects could contribute to this phenotype in a more indirect manner, such as post translational modifications of stably expressed proteins that activate or inactivate certain functional activities important for the completion of cytokinesis of newly formed zoites and/or egress of infective tachyzoites. However, characterizing the formation of this drug-induced stage and identifying conserved molecular signatures of the BKI-induced parasitostatic response and shared stress pathways can offer new insights into the biology and persistence of cyst-forming apicomplexans.

## 4. Materials and Methods

### 4.1. Cell Culture Media, Biochemicals, and BKI-1708

Culture medium is from Gibco-BRL (Zürich, Switzerland), and biochemicals are from Sigma (St. Louis, MO, USA). BKI-1708 was originally synthesized in the Department of Biochemistry of the University of Washington, USA, and scaled up by WuXi Apptec Inc., Wuhan, China, to >98% purity by LC/MS-MS and NMR and shipped as powder [54]. For in vitro studies, stock solutions of 20 mM were prepared in dimethyl-sulfoxide (DMSO) and stored at −20 °C.

### 4.2. Host Cells and Parasites

Human foreskin fibroblasts (HFFs; PCS-201-010™) were maintained as previously described [19]. The strains used in this study were *T. gondii* ME49, *N. caninum* Spain-7 isolate (NcSpain-7) and *B. besnoiti* Bblis. They were maintained in HFF as previously described in [4].

### 4.3. BKI-1708 Treatments and Transmission Electron Microscopy (TEM)

For TEM, HFFs were grown to confluence in T25 flasks in culture medium for three days at 37 °C/5% CO_2_ and were infected with 1 × 10^6^ *T. gondii* ME49, *N. caninum* Nc-Sp7 or *B. besnoiti* Bblis14 tachyzoites. At 4 h post-infection, the medium was supplemented with 2.5 µM BKI. The medium was removed after 24 h for untreated cultures, and at days 4 and 6 of continuous treatment, with BKI-1708 supplemented medium being renewed every second day. After 4 and 6 days of exposure to BKI-1708, cultures were washed once with 100 mM sodium cacodylate, pH 7.3, and fixation was carried out in 100 mM cacodylate buffer containing 2% glutaraldehyde for 10 min. Then, adherent cells were carefully removed with a cell scraper and transferred into a tube. After another 2–4 h of fixation at room temperature, samples were centrifuged and post-fixed in 2% cacodylate buffer containing 2% osmium tetroxide for 2 h. Following several washes in distilled water, specimens were dehydrated through a graded series of ethanol (30, 50, 70, 90 and 3 × 100%), and resuspended in Epon 812 epoxy resin (Sigma (St. Louis, MO, USA) as previously described [14]. After three changes in Epon resin, polymerization was carried out at 60 °C overnight. Sections of 80 nm thickness were cut on an ultramicrotome (Reichert and Jung, Vienna, Austria) and were transferred onto formvar–carbon-coated 200 mesh nickel grids (Plano GmbH, Marburg, Germany). They were stained with Uranyless^®^ and lead citrate (Electron Microscopy Sciences, Hatfield, PA, USA), and specimens were inspected on a FEI Morgagni TEM equipped with a Morada digital camera system (12 Megapixel) operating at 80 kV.

### 4.4. Reversion and Long-Term Treatment Assays

The 5 × 10^5^ HFFs were grown to confluence in T25 flasks. These monolayers were then infected with 5 × 10^6^ tachyzoites of TgME49, Nc-Sp7, or Bblis14 tachyzoites. Cultures were washed with phosphate-buffered saline (PBS) at 1 h post-infection and were exposed to 2.5 µM BKI-1708 during 5 days at 37 °C/5% COBKI-supplemented media was renewed on the third day of treatment, and control cultures were left untreated. After 5 days of continuous treatment, cultures were washed with PBS and further maintained in fresh medium without drug. Regrowth of parasites cultures was monitored every day by conventional light microscopy until host cell lysis (lysis plaque formation) and extracellular tachyzoites were visible. To assess the effects of a more prolonged treatment on *T. gondii*, 5 × 10^5^ HFFs were grown to confluence in T25 flasks and subsequently infected with 5 × 10^6^ *T. gondii* ME49 tachyzoites. One hour post-infection, cultures were washed with PBS and the medium was supplemented with 2.5 µM BKI-1708, followed by culture at 37 °C/5% CO_2_ for 30 days. Cultures were left in BKI-supplemented medium and monitored every second day with conventional light microscopy.

### 4.5. Comparative Proteomics

Semi-confluent HFF monolayers were maintained in T75 cell culture flasks, and were infected with 1 × 10^7^ TgME49, Nc-Spain7 or Bblis14 tachyzoites. At 4 h post-infection, treatment with 2.5 µM BKI-1708 was initiated, control cultures were not exposed to BKI. Three biological replicates were used for each. All non-treated control cultures were maintained at 37 ◦C/5% CO_2_ during 3 days, BKI-1708 treated cultures during 5 days (BKI-1708). Subsequently, the infected monolayers were washed twice with PBS, followed by removal of infected cells with a rubber cell scraper and resuspension in PBS followed by centrifugation (15 min, 1000× *g*, 4 °C). Cell pellets were lysed in 100 µL 8 M urea/100 mM Tris/HCl pH 8/cOmpleteTM protease inhibitor cocktail (Roche Diagnostics, Rotkreuz, Switzerland) by incubation for 15 min at RT followed by 15 min in an ultrasonic water bath. Proteins were reduced and alkylated with 10 mM DTT for 30 min at 37 °C and 50 mM iodoacetamide for 30 min at 37 °C. Proteins were precipitated at −20 °C by addition of 5 volumes of cold acetone and incubation at −20 °C overnight. All liquid was carefully removed, and the pellet dried in ambient air for 15 min before reconstitution of proteins in 8 M urea, 50 mM Tris-HCl pH 8.0 to an approximate protein concentration of 2 mg/mL. Effective protein concentrations were determined by Bradford assay on 1:10 diluted samples. A total of 10 µg of protein was diluted in 0.6 M urea, 50 mM Tris-HCl (pH 8.0) and subjected to enzymatic digestion with LysC (1:100) for 2 h at 37 °C, followed by overnight digestion with trypsin (1:50) at ambient temperature.

Samples were subjected to proteomic analysis using shotgun nano liquid proteomics on a timsTOF HT mass spectrometer with PASEF coupled to a nanoElute2 UPLC (Bruker, Billerica, MA, USA) applying a 30 min peptide separation gradient on a PepSep column (150 μm × 15 cm, Bruker) coupled with a captive ion source to the mass spectrometer. Data were acquired via the DDA method employing eight PASEF ramps with an ion mobility range from 0.75 to 1.35 1/k0, in the *m*/*z* range between 100 and 1700 u and a cycle time of 0.95 s at an acquisition rate of 300 Hz. For DIA 32 isolation windows were set, each 26 *m*/*z* wide, with a 1 *m*/*z* overlap between adjacent windows. These isolation windows corresponded to an ion mobility range of 0.75–1.36 V s/cm. Both TIMS accumulation and separation times were set at 100 ms [55].

The mass spectrometry data were searched and quantified with Spectronaut (Biognosys, Schlieren, Switzerland) version 19.0.240606.62635 in the hybrid directDIA+ (Deep) with factory settings. Search parameters included Acetyl (Protein N-term) and Oxidation (M) as variable modifications (5 max variable modifications), Carbamidomethyl (C) as fixed modification, and Trypsin/P (max 2 missed cleavages) as digestion enzyme. The Tg+host, Nc+host and Bb+host samples were searched respectively against the ToxoDB-55 [56]. *T. gondii* ME49 annotated protein sequences, a concatenation of swissprot and uniport *N. caninum* (strain Liverpool) sequences (https://www.uniprot.org; release July 2024; accessed 1 September 2025), and a concatenation of swissprot and ToxoDB-68 *B. besnoiti* sequences. Common contaminants were added in all three databases. Protein groups with less than two peptides per group were excluded. A leading protein was chosen per protein group on the basis of best coverage, and the IBAQ (intensity-based absolute quantification) values calculated by Spectronaut are reported here with respect to the leading protein. A relative abundance (rAbu) was calculated so that the sum of rAbu were 1,000,000 for every sample. In addition to Spectronaut’s SN abundance values, ion-based quantification values IQ from the *iq* R package [57] were calculated for the *N. caninum* and the *B. besnoiti* datasets.

Protein groups not flagged as potential contaminants and for which we had at least two detections in at least one of the replicate groups were considered for differential expression. Missing values were then imputed in the following manner: if there was at most two non-zero values in the replicate group then the missing values were imputed by a left-censored method; this was done by drawing random values from a Gaussian distribution of width 0.3 × sample standard deviation centered at the sample distribution mean minus 2.5 × sample standard deviation. Any remaining missing values were imputed by the Maximum Likelihood Estimation (MLE) method [58]. Differential expression tests were performed with the moderated *t*-test of the R limma package [59]. The Benjamini and Hochberg [60] method was then applied to correct for multiple testing. The criterion for statistically significant differential expression was that the largest accepted adjusted *p*-value reaches 0.05 asymptotically for large absolute values of the log2 fold change and tends to 0 as the absolute value of the log2 fold change approaches 1 (with a curve parameter of 0.1× overall standard deviation). Proteins consistently significantly differentially expressed through 20 imputation cycles were flagged accordingly [61]. Similar imputation cycles and significance tests were performed on the IQ values for the *N. caninum* and the *B. besnoiti* datasets. In these two cases an additional significance criterion was the agreement between the significance of IQ and SN values.

### 4.6. Fluorescence Microscopy

For immunofluorescence studies, 2 × 10^4^ HFFs were grown on glass coverslips in 24-well plates at 37 °C and 5% CO_2_ for three days, and then infected with 2 × 10^4^ TgMe49, NcSpain-7 or Bblis14 tachyzoites. Four hours after infection, the medium was replaced and supplemented with 2.5 μM BKI-1708, or medium without compound. Cultures were maintained at 37 °C and 5% CO_2_ for a maximum of 9 days, with BKI-1708 supplemented medium being changed every second day. On days 2, 6 and 9, cells were fixed in 3% paraformaldehyde in PBS (pH 7.2) for 10 min, and permeabilized in a 1:1 solution of pre-cooled methanol/acetone at −20 °C. Following rehydration, nonspecific binding sites were blocked in PBS/3% bovine serum albumin (BSA) overnight at 4 °C. For immunofluorescence double staining, primary and secondary antibodies were diluted in PBS containing 0.3% BSA and added sequentially to the samples during 30 min each. The primary antibodies used in this study are listed in Table 5, corresponding secondary antibodies were tetramethyl-rhodamine-isothiocyanate (TRITC)-conjugated anti-mouse, FITC-conjugated anti-rabbit, and TRITC-conjugated anti-bovine serum all at a dilution of 1. Following antibody staining, samples were thoroughly washed in PBS and were mounted in, H-1200 Vectashield mounting medium with DAPI (4′,6-diamidino-2-phenylindole; Vector Laboratories, Inc. Burlingame, CA, USA), and viewed on a Nikon E80i fluorescence microscope. Processing of images was performed using ImageJ software (version 1.54p) from National Institutes of Health, Bethesda, MD, USA [62].

## 5. Conclusions

The consistent formation of baryzoites across *T. gondii*, *N. caninum*, and *B. besnoiti* and other apicomplexans in response to BKI treatment points to a conserved and regulated stress response, although with species-specific differences. Baryzoites are defined as a distinct drug-induced dormant stage in cyst-forming parasites, displaying tachyzoite and bradyzoite features, triggered and maintained by drug pressure in vitro. They are characterized by the presence of numerous nuclei resulting from continuous division, with an accumulation of newly formed daughter zoites within the host cell due to impaired cytokinesis. The existence of baryzoites in vivo is uncertain, since no specific marker has been identified to date. However, similar multinucleated forms have been described in *T. gondii* upon treatment with diclazuril, a triazinone derivative that is targeting enzymes such as components of the mitochondrial respiratory chain and dihydrofolate reductase [68]. Other repurposed compounds, such as the dopamine receptor pinozode and the estrogen antagonist tamoxifen also caused the formation of multinucleated forms of tachyzoites in vitro [69]. Thus, the formation of a heavily enlarged parasitic stage that ensures survival in response to increased drug concentrations during prolonged periods of time might be more common than previously anticipated. Baryzoites grow exceptionally large by undergoing multiple rounds of nuclear division without forming complete daughter cells, reminiscent of schizonts, as seen in other Apicomplexa such as *Plasmodium* spp. Although these stages of asexual reproduction in *Toxoplasma gondii* are traditionally called meronts, the multi-nuclear baryzoite-phenotype suggests a functional resemblance to schizonts. To what extent baryzoites resemble the merogony stage that occurs in the cat intestine, during which the parasite undergoes several rounds of DNA replication and mitosis without immediately dividing, needs to be further investigated.

## Figures and Tables

**Table 1:** Summary of protein abundance changes in *T. gondii* ME49, *N. caninum* Nc-Sp7, and *B. besnoiti* BbLis14 baryzoites compared to untreated tachyzoites.

	*T. gondii* Baryzoites	*N. caninum* Baryzoites	*B. besnoiti* Baryzoites	Commonly DE Proteins
Total detected proteins	2670	1461	2755	19
Proteins at higher abundance	153 (5.7%)	15 (1%)	39 (1.4%)	3
Proteins at lower abundance	290 (10.9%)	114 (7.8%)	389 (14.1%)	16

**Table 2:** List of the sixteen proteins found at lower abundance in *T. gondii, N. caninum* and *B. besnoiti* baryzoites compared to untreated parasites (tachyzoites). Proteins are identified by ORF IDs listed in ToxoDB. The arithmetic mean of relative abundances of proteins analyzed in triplicate is shown with the correspondent standard deviation, rounded to the nearest tenth (rAbu ± SD).

Annotation in *N. caninum*	*N. caninum*	rAbu ± SD	Orthologue *T. gondi*	rAbu ± SD	Orthologue *B. besnoiti*	rAbu ± SD
Profilin	NCLIV_000610	140 ± 3.4	TGME49_293690	1110.4 ± 314.7	BESB_082700	311.2 ± 21.5
Calmodulin, putative	NCLIV_001300	110 ± 14.4	TGME49_305050	407.4 ± 42.3	BESB_025690	193.8 ± 6.7
Yml024wp-like protein, related	NCLIV_002780	49 ± 11	TGME49_207840	541.4 ± 83	BESB_022530	209.8 ± 16.1
40S ribosomal protein S28, related	NCLIV_003680	65.5 ± 3.4	TGME49_209290	186.9 ± 58.4	BESB_023540	448 ± 109.1
40S ribosomal protein S6	NCLIV_012120	210.6 ± 22	TGME49_210690	810.2 ± 246.2	BESB_016090	594.5 ± 73.9
40S ribosomal protein S15	NCLIV_013320	202.5 ± 27.7	TGME49_213350	474 ± 78.7	BESB_046950	753 ± 57.7
60S ribosomal protein L23a	NCLIV_015410	60.7 ± 9.5	TGME49_238010	467.9 ± 157	BESB_081300	168 ± 14
40S ribosomal protein S7	NCLIV_015880	144.5 ± 23.1	TGME49_239100	1705 ± 32.7	BESB_082060	465.3 ± 42
60S ribosomal protein L13	NCLIV_024870	89.6 ± 11.1	TGME49_263050	479.2 ± 119.3	BESB_001210	535.5 ± 40.8
30S ribosomal protein S12, related	NCLIV_030490	41.3 ± 14.1	TGME49_229670	698.2 ± 173.9	BESB_083100	266.4 ± 105.6
30S ribosomal protein S15P/S13e, related	NCLIV_036280	92.6 ± 22	TGME49_270380	712.8 ± 169	BESB_053520	369.6 ± 32.7
60S ribosomal protein L32, related	NCLIV_038780	125.6 ± 23.4	TGME49_267400	586.4 ± 139.1	BESB_050430	510.1 ± 51.5
40S ribosomal protein S24	NCLIV_052380	100 ± 12.4	TGME49_215460	310 ± 75.3	BESB_034520	400.2 ± 20.5
Ribosomal protein	NCLIV_052390	47 ± 12.2	TGME49_215470	2373 ± 450	BESB_034510	301.4 ± 28.7
60S ribosomal protein L6	NCLIV_056680	190.6 ± 7.9	TGME49_313390	268.4 ± 59.4	BESB_074380	677.3 ± 61.8
60S acidic ribosomal protein P0	NCLIV_061830	38.2 ± 12.7	TGME49_218410	291 ± 89	BESB_039290	99.3 ± 8.1

**Table 3:** SRS-domain surface proteins expressed at low and high abundance in *T. gondii*, *N. caninum* and *B. besnoiti* baryzoites. ORF IDs correspond to those listed in ToxoDB. Bradyzoite-specific SRS proteins are marked with an asterisk (*).

**SRS-domain surface antigen proteins expressed at lower abundance in baryzoites**					
***T. gondii*** **ToxoDB ORF**	**Annotation *T. gondii***	***N. caninum*** **ToxoDB ORF**	**Annotation *N. caninum***	***B. besnoiti*** **ToxoDB ORF**	**Annotation *B. besnoti***
0 proteins		BN1204_035215	SRS-domain	BESB_033560	SAG-related sequence
		1 protein		BESB_034140	SAG-related sequence
				BESB_034280	SAG-related sequence
				BESB_038370	SAG-related sequence
				BESB_050150	SAG-related sequence
				BESB_053070	SAG-related sequence
				BESB_057290	SAG-related sequence
				BESB_057360	SAG-related sequence
				BESB_057390	SAG-related sequence
				BESB_057430	SAG-related sequence
				BESB_057440	SAG-related sequence
				BESB_061470	SAG-related sequence
				BESB_084440	SRS28
				BESB_040500	SRS57
				BESB_059740	SRS67
				15 proteins	
**SRS-domain surface proteins expressed at higher abundance in baryzoites**					
***T. gondi*** **ToxoDB ORF**	**Annotation *T. gondii***	***N. caninum*** **ToxoDB ORF**	**Annotation *N. caninum***	***B. besnoiti*** **ToxoDB ORF**	**Annotation *B. besnoti***
TGME49_222370	SRS13	0 proteins		BESB_035050	SAG-related sequence
TGME49_320230	SRS15C			BESB_044470	SAG-related sequence
TGME49_320170	SRS16E			BESB_079200	SAG-related sequence
TGME49_301150	SRS19B			BESB_043640	SAG-related sequence
TGME49_301160	SRS19C			BESB_001090	SAG-related sequence
TGME49_301180	SRS19F			BESB_050090	SAG-related sequence
TGME49_273130	SRS30A			BESB_043590	SAG-related sequence
TGME49_280570	SRS35A *			BESB_050020	SAG-related sequence
TGME49_292260	SRS36B			BESB_050210	SAG-related sequence
TGME49_292280	SRS36D			BESB_049990	SAG-related sequence
TGME49_267160	SRS38D			BESB_081950	SRS23
TGME49_281930	SRS39			11 proteins	
TGME49_224770	SRS40D				
TGME49_264660	SRS44 *				
TGME49_207010	SRS48K				
TGME49_207130	SRS49A *				
TGME49_207160	SRS49D *				
TGME49_315410	SRS53F				
18 proteins					

**Table 4:** List of the ten most abundant proteins detected in *Toxoplasma gondii* baryzoites. Proteins are ranked according to the arithmetic mean of triplicates’ relative abundance (rAbu), with values presented as mean ± standard deviation (SD).

ToxoDB ORF	Annotation	rAbu	SD
TGME49_218260	histone H3.3	34,537	±6394
TGME49_300200	histone H2AZ	25,146	±5105
TGME49_225410	histone H3 centromeric CENH3	13,850	±2712
TGME49_249240	calmodulin, putative	4685	±1467
TGME49_248700	alveolin domain containing intermediate filament IMC12	3150	±503
TGME49_222220	alveolin domain containing intermediate filament IMC7	2556	±361
TGME49_226570	hypothetical protein	2417	±399
TGME49_270240	MAG1	2334	±874
TGME49_280570	SAG-related sequence SRS35A	1506	±636
TGME49_236950	hypothetical protein	1261	±337

**Table 5:** Primary antibodies used for immunofluorescence (IF). Host species, antibody type, target, origin and working dilutions are indicated. * *Dolichos Biflorus* Agglutinin (DBA) is a lectin conjugated to fluorescein isothiocyanate (FITC).

Parasite	Antigen	Host	Type	Target	Origin	Dilution
*Toxoplasma*	TgSAG1	mouse	monoclonal	tachyzoite surface antigen	[14]	1:100
*Neospora*	NcSAG1	mouse	monoclonal	tachyzoite surface antigen	[14]	1:1000
*Toxoplasma* and *Neospora*	TgIMC1	rabbit	polyclonal	inner membrane complex **1**	[63]	1:500
*Toxoplasma* and *Neospora*	TgBAG1	rabbit	polyclonal	bradyzoite antigen (HSP30)	[64]	1:200
*Toxoplasma* and *Neospora*	NTPase	rabbit	polyclonal	nucleoside diphosphate hydrolase	[65]	1:300
*Toxoplasma* and *Neospora*	115 kDa cyst wall antigen	mouse	monoclonal	cyst wall protein	[66]	1:300
*Besnoitia*	Anti-*Besnoitia*	bovine, naturally infected	polyclonal	*Besnoitia* spp. tachyzoites	[67]	1:1000
*Toxoplasma*	DBA *		lectin	N-acetyl-D-galactosamine (GalNAc)	[33]	1:300

## Data Availability

The original contributions presented in this study are included in the article/Supplementary Material. Further inquiries can be directed to the corresponding authors.

## Supplementary Materials

The following supporting information can be downloaded at: https://www.mdpi.com/article/10.3390/ijms27062914/s1.
