# Supplementary figures and images for: Bumped Kinase Inhibitor BKI-1708 Interferes in Cytokinesis and Drives Baryzoite Conversion in the Cyst-Forming Apicomplexan Parasites Toxoplasma gondii, Neospora caninum and Besnoitia besnoiti
Source: Int J Mol Sci. 2026 Mar 23;27(6):2914. doi: 10.3390/ijms27062914 (PMC13027286; doi:10.3390/ijms27062914)

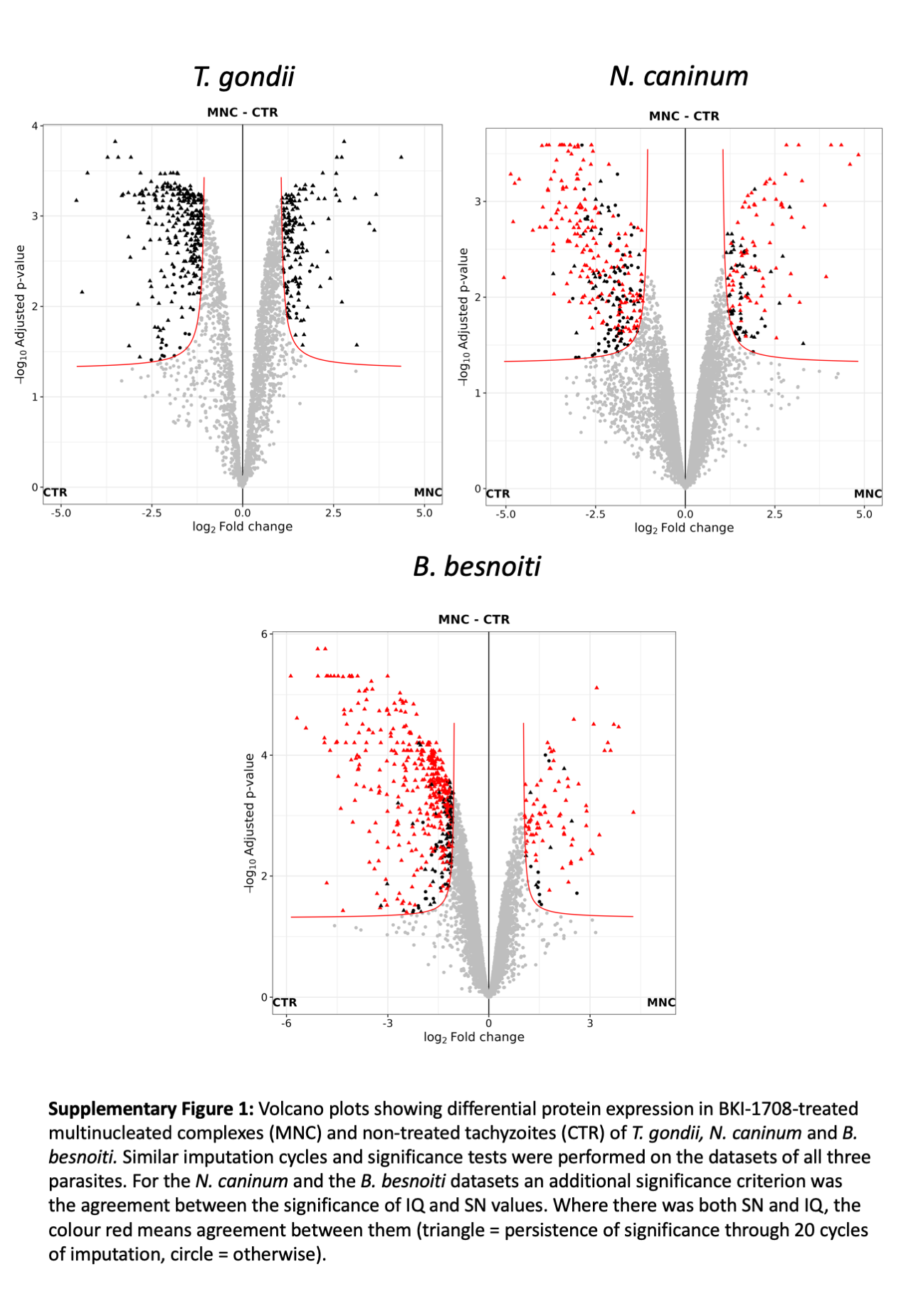

Supplement: Supplementary file 1 [file ijms-27-02914-s001.zip › Supplementary tables and figure 2/Supplementary Figure 1.tiff]

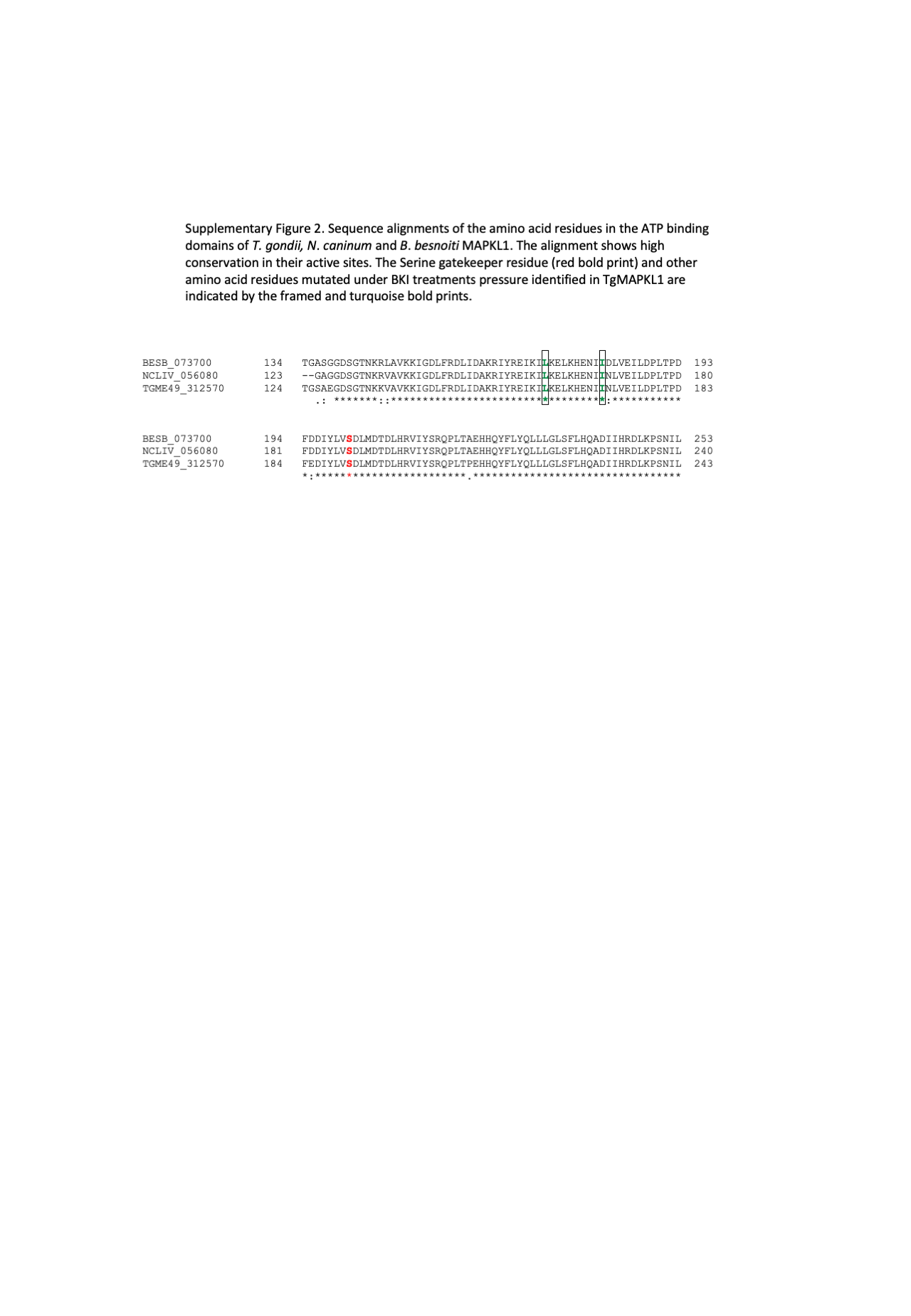

Supplement: Supplementary file 1 [file ijms-27-02914-s001.zip › Supplementary tables and figure 2/Supplementary Figure 2.tiff]
